# Parietal cells—new perspectives in glomerular disease

**DOI:** 10.1007/s00441-017-2600-5

**Published:** 2017-03-30

**Authors:** Laura Miesen, Eric Steenbergen, Bart Smeets

**Affiliations:** Department of Pathology, RIMLS, RIHS, Radboud University Medical Center, Geert Grooteplein 24, 6525 GA Nijmegen, The Netherlands

**Keywords:** Parietal epithelial cell, Glomerulus, Focal and segmental glomerulosclerosis, Crescentic glomerulonephritis, Podocyte regeneration

## Abstract

In normal glomeruli, parietal epithelial cells (PECs) line the inside of Bowman’s capsule and form an inconspicuous sheet of flat epithelial cells in continuity with the proximal tubular epithelial cells (PTECs) at the urinary pole and with the podocytes at the vascular pole. PECs, PTECs and podocytes have a common mesenchymal origin and are the result of divergent differentiation during embryogenesis. Podocytes and PTECs are highly differentiated cells with well-established functions pertaining to the maintenance of the filtration barrier and transport, respectively. For PECs, no specific function other than a structural one has been known until recently. Possible important functions for PECs in the fate of the glomerulus in glomerular disease have now become apparent: (1) PECs may be involved in the replacement of lost podocytes; (2) PECs form the basis of extracapillary proliferative lesions and subsequent sclerosis in glomerular disease. In addition to the acknowledgement that PECs are crucial in glomerular disease, knowledge has been gained regarding the molecular processes driving the phenotypic changes and behavior of PECs. Understanding these molecular processes is important for the development of specific therapeutic approaches aimed at either stimulation of the regenerative function of PECs or inhibition of the pro-sclerotic action of PECs. In this review, we discuss recent advances pertaining to the role of PECs in glomerular regeneration and disease and address the major molecular processes involved.

## Podocyte regeneration by parietal epithelial cells

Podocytes play a key role in maintaining the glomerular filtration barrier and podocyte dysfunction or podocyte loss is the determining factor for the initiation and progression of glomerulosclerosis (Kriz et al. 1998). Since podocytes can be isolated and cultured from the urine of healthy individuals, we clearly lose podocytes even under normal physiological circumstances. The loss of podocytes is accelerated in glomerular diseases such as glomerulonephritis and diabetic glomerulopathy. High losses can lead to insufficient podocyte numbers that are no longer compatible with long-term glomerular survival. We therefore urgently need to find ways to prevent podocyte loss and to replace lost podocytes. Podocytes themselves are highly differentiated postmitotic cells that cannot divide and have therefore no capacity for regeneration. Pushing podocytes beyond cell cycle arrest leads to polyploidy and eventually to death. The same principle applies to all other differentiated epithelial cells of the body. Therefore, research has aimed at identifying other cell sources that might act as podocyte progenitors and be responsible for the regeneration or replacement of lost podocytes. During the last 8 years, parietal epithelial cells (PECs) have come into focus as putative podocyte progenitors. Both the transition from PECs to podocytes and the functional replacement of podocytes by PECs have been studied. Until now it has remained unclear whether podocytes are effectively replaced by PECs or by cells from any other sources.

Recent studies have indicated that, during human life, a gradual decrease occurs in the total number of podocytes within the kidney (Hodgin et al. 2015; Puelles et al. 2016). These studies have shown that the human glomerulus contains approximately 600 podocytes in roughly 1 million nephrons per kidney and that we lose about 0.34 % of the podocytes per glomerulus per year (∼1-2 podocytes/glomerulus/year; Hodgin et al. 2015). This loss is accompanied by a gradual increase in the glomerular tuft volume. In the aging kidney both the loss of podocytes and the increasing tuft volume lead to a marked podocyte depletion that might be associated with age-related (global) glomerulosclerosis. From these studies, one can conclude that the assumed process of podocyte replacement is not sufficient to prevent podocyte depletion in the aging kidney. However, this does not rule out that podocyte replacement by podocyte progenitors does indeed occur and prevents an even faster decrease in podocyte number than that currently measured.

Most podocytes are formed during glomerular development. Remarkably, a recent study indicated that the number of podocytes increases during glomerular growth and maturation in the early years after birth, raising questions about the origin of these new podocytes (Puelles et al. 2015). A possible explanation for this may come from earlier investigations. Even in 2009, Appel and co-workers showed by using lineage tracing of PECs that, in immature growing mouse glomeruli, podocytes were recruited from the cells lining Bowman’s capsule (Appel et al. 2009). Initially, this was believed to be the result of PEC transdifferentiation. However, these cells could well have been already committed to become podocytes as the cells traced on Bowman’s capsule already exhibit the expression of the podocin promotor and the expression of various podocyte markers, as suggested by Berger et al. (Berger et al. 2014).

Whether podocytes are regenerated in mature mammalian kidneys after injury remains controversial. Several studies have argued in favor of podocyte replacement by PECs based on the finding of the increased co-expression of podocyte- and PEC-specific markers, either on cells lining Bowman’s capsule or on the glomerular tuft. However, clearly, the transcriptional program of PECs is closely related to that of podocytes and the co-expression of podocyte- and PEC-specific markers by podocytes and/or PECs might represent aberrant marker expression triggered by underlying pathological conditions (Guhr et al. 2013; Sakamoto et al. 2014). In addition, an assessment of whether podocytes are truly lost and replaced is difficult. An accurate estimation of podocyte numbers is problematic and laborious and even state-of-the-art methods rely on podocyte-specific markers, the expression of which may change in states of disease. Loss of marker expression does not necessarily reflect loss of podocytes but may be associated with podocyte injury and loss of function. Similarly, an increase of cells expressing podocyte-specific markers may simply reflect the restored expression of the marker.

The technique of lineage/fate tracing has been used largely to overcome the limitation of marker studies. By using this technique, podocyte regeneration has been studied in experimental models of aging, podocyte injury and glomerular hypertrophy. The majority of these studies have not been able to provide evidence for the recruitment of podocytes from PECs (Berger et al. 2014; Sakamoto et al. 2014; Wanner et al. 2014). However, two recent studies with PEC-specific reporter systems found evidence suggesting podocyte regeneration in models of acute podocyte depletion. Eng et al. (2015) demonstrated that, in a model of acute podocyte depletion, PECs undergo two different phenotypic changes. Initially, PECs become activated, expressing CD44 and phosphorylated extracellular-signal-regulated kinase-1 (Erk-1). Later, a sup-population of the traced PECs expresses podocyte markers without Erk-1 (Eng et al. 2015). Lasagni et al. (2015) demonstrated that, in animals that have attained disease remission after the previous induction of acute podocyte injury, podocytes are regenerated from PECs. In these animals, the genetic PEC reporter signal has been traced in cells that, based on their location, phenotype and marker expression, resemble podocytes (Lasagni et al. 2015).

Taken together, the data concerning effective podocyte regeneration and the role of PECs in this process are still confounding and more animal studies are needed. In humans, difficulties will be experienced in establishing podocyte regeneration, as this would require the accurate assessment of podocyte numbers in successive renal biopsies and would also require information concerning podocyte loss.

## Stimulating podocyte regeneration

Podocyte regeneration by PECs depends on the successful transdifferentiation of PECs towards a podocyte phenotype. In the last few years, various pathways have been identified as playing a role in this cell transition. New insights demonstrate an important role for the retinoic acid pathway. Retinoic acid is a metabolite of vitamin A (retinol) and acts by binding to the retinoic acid receptor (RAR), which in turn binds with the retinoid X receptor (RXR) in DNA regions called retinoic acid response elements (RAREs). The retinoic acid receptor complex mediates the transcription of various sets of genes controlling differentiation in a variety of cell types. In the glomerulus, retinoic acid mediates the differentiation of podocytes (Suzuki et al. 2003). Moreover, in PECs, podocyte differentiation markers are up-regulated when the cells are treated with retinoic acid. In rats with experimental membranous nephropathy and in a mouse model of glomerulosclerosis, treatment with all-trans retinoic acid has been shown to result in an increase in number of cells expressing podocyte and PEC markers and an increased total number of podocytes (Zhang et al. 2012). However, Peired et al. (2013) showed that, in albuminuric kidneys, albumin sequesters retinoic acid in Bowman’s space. For the treatment of humans, this would necessitate the use of very high and toxic doses of retinoic acid (Peired et al. 2013). A recent study from the same group showed that the effects of retinoic acid can be enhanced: the glycogen synthase kinase 3 (GSK3) inhibitor BIO (6-bromo-indirubin-3-oxime) acts by increasing the binding of retinoic acid to its specific RARE elements, thus enhancing the effect of retinoic acid. Lasgnai et al. (2015) proposed that the use of BIO retinoic acid improves the transdifferentiation of PECs to podocytes and avoids toxicity.

During the last few years, several studies have presented evidence for the importance of microRNAs. MicroRNAs are short non-coding RNAs that are involved in the post-transcriptional regulation of gene expression. With regard to glomerular epithelial cells, an important role for microRNA (miR)-193a has been detected in podocyte differentiation and PEC to podocyte transdifferentiation. In podocytes, miR-193a induces the down-regulation of WT-1 resulting in podocyte injury and focal segmental glomerulosclerosis (Gebeshuber et al. 2013). In cultured human immortalized PECs, the stable knockdown of miR-193a has been demonstrated to induce a podocyte-like morphology and marker expression of WT-1, podocalyxin, synaptopodin, α-actinin-4 and nephrin. PEC marker expression decreases, e.g., Pax8, claudin-1 and UCH-L1 (Kietzmann et al. 2015). Similar findings have been made in vivo in mice. The inhibition of miR-193a by complementary locked nucleic acids results in the up-regulation of the podocyte proteins synaptopodin and WT-1. Conversely, the overexpression of miR-193a in vivo leads to the up-regulation of PEC markers and the loss of podocyte markers. Interestingly, the inhibition of miR-193a in a mouse model of crescentic glomerulonephritis (CrGN) results in a decrease in crescent formation and proteinuria.

## PECs: a key player in extracapillary proliferation and sclerosis

Following capillary wall or podocyte injury, extracapillary proliferative lesions may develop. In human glomerular disease extracapillary proliferation is considered prognostically adverse as it leads to scarring. Severe glomerulonephritis, such as anti glomerular basement membrane disease (anti-GBM disease) and anti neutrophil cytoplasmic antibodies associated disease (ANCA-disease), is typically associated with extensive extracapillary proliferation called cellular crescents. Many other forms of glomerulonephritis, such as IgA nephropathy, frequently have a more chronic clinical course. In these conditions, “active” extracapillary proliferative lesions and older scar lesions are typically simultaneously present in renal biopsies. Extracapillary proliferative lesions are also seen in focal and segmental glomerulosclerosis (FSGS). FSGS is considered a non-inflammatory process resulting from podocyte injury. In FSGS, extracapillary proliferative lesions are called pseudocrescents because of their non-inflammatory nature. FSGS occurs as a primary (podocyte) disease but importantly, almost all other glomerular diseases give rise to secondary FSGS lesions that, for a large part, determine prognosis. Thus, extracapillary proliferation leading to scarring plays a role in virtually all human glomerular diseases. A better understanding of the nature of these lesions is therefore extremely important.

**Fig. 1:**
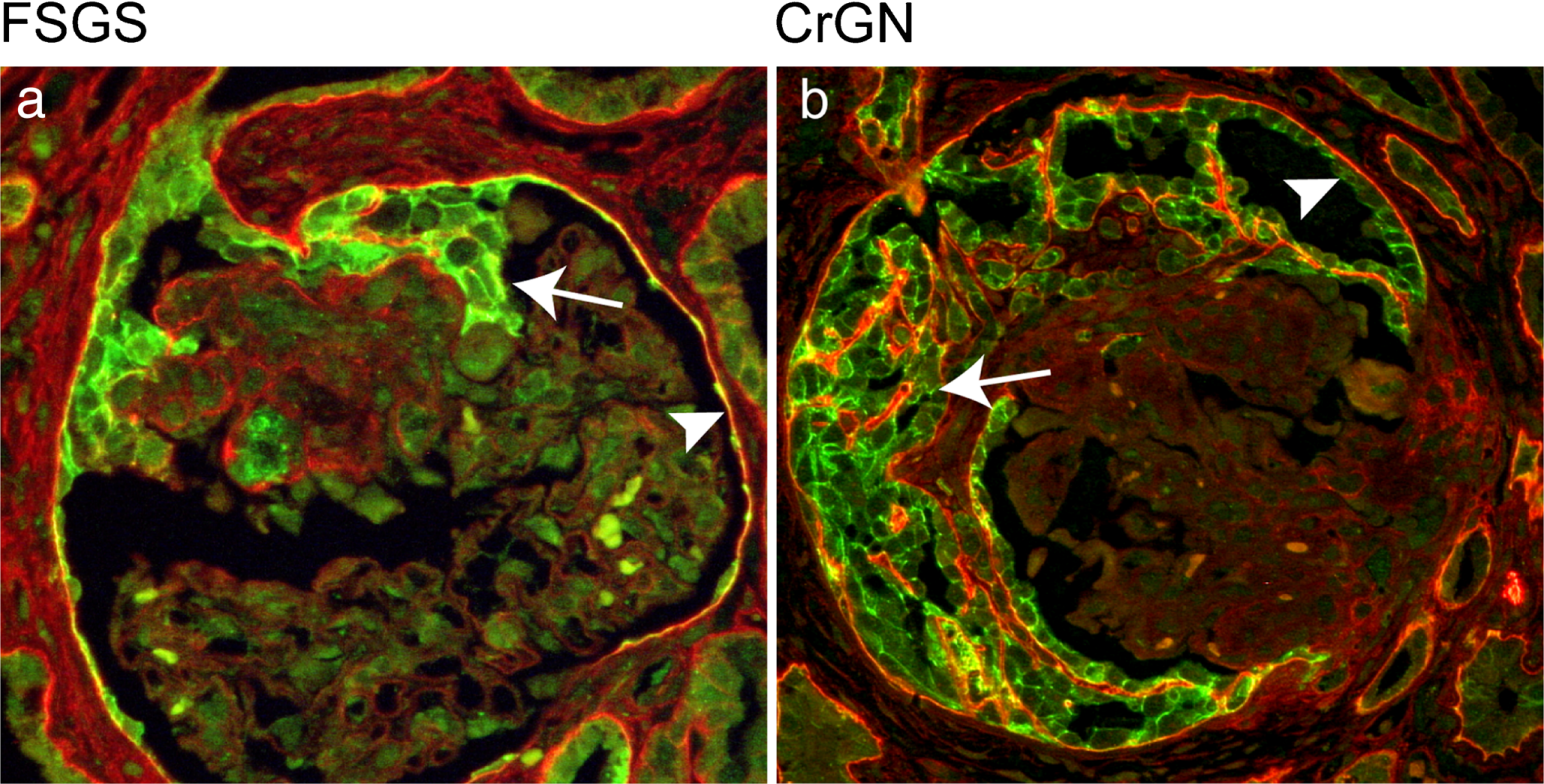
Extracapillary proliferative lesions consisting in parietal epithelial cells (PECs) in glomeruli of patients with focal and segmental glomerulosclerosis (*FSGS*; **a**) and crescentic glomerulonephritis (*CrGN*; **b**). Double immunostaining for claudin-1 expressed by PECs (*green*, *arrowheads*) and specific heparan sulfates in the extracellular matrix (*red*). Note marked hyperplasia of claudin-1-positive PECs (*arrows*) in both FSGS and CrGN

Historically, “true” crescents were considered to result from the proliferation of PECs secondary to capillary wall necrosis and fibrinous exudate. In fact, glomerular capillary wall rupture was shown to be sufficient to induce PEC proliferation by exposure to serum only (Ryu et al. 2012). In contrast, pseudocrescents were thought to be of podocyte origin, based on the finding that cell increase often appeared to be confined to the glomerular tuft, without apparent connection to the PEC layer. Subsequently, several lines of evidence firmly established that extracapillary hypercellularity in FSGS was the result of PEC proliferation. Initially, marker studies in a mouse model of collapsing FSGS showed that proliferating epithelial cells stained for parietal markers, produced PEC-specific extracellular matrix and were negative for podocyte markers. These marker studies were not entirely conclusive, as it could not be excluded that proliferating cells had indeed acquired a PEC phenotype as a result of the pathological condition and were of a different (presumably podocyte) origin. The definitive answer came by means of work carried out on transgenic mice in which the PEC or the podocyte had been irreversibly genetically labeled. Both in the model of collapsing FSGS and in the mouse anti-GBM model of CrGN, proliferating epithelial cells were shown to be derived from PECs and to express the activation marker CD44 (Smeets et al. 2009). In the human situation, the labeling of cells to investigate their fate in disease is not possible but marker studies in human FSGS are consistent with the findings in mice (Fig. 1). As for the mechanism that leads to cellular FSGS lesions, initial insight came from detailed three-dimensional analysis of glomeruli from a patient with the recurrence of collapsing FSGS after transplantation. The suggestion was made that activated cells with a PEC phenotype migrate onto the glomerular tuft via cellular bridges between the tuft and Bowman’s capsule (Dijkman et al. 2005). More insight into the mechanism of extracapillary hypercellularity and scarring came from a detailed marker study of secondary FSGS lesions in 11 different human glomerulopathies. This investigation made use of triple immunostaining (podocyte marker synaptopodin with PEC marker annexin 3 or CD44 and a marker for PEC-specific extracellular matrix) in combination with light microscopy. In this way, a consistent sequence of events in the formation of all secondary FSGS lesions can be detected: (1) the earliest lesions consist in cellular bridges between Bowman’s capsule and the glomerular tuft formed by parietal cells that deposit PEC matrix on the tuft; (2) subsequently, PECs migrate onto the glomerular tuft and small matrix adhesions are formed (early sclerotic lesions); (3) in advanced sclerotic lesions, a larger part of the tuft is covered by PECs that deposit PEC-specific extracellular matrix. The more cellular the FSGS lesions are, the more CD44-positive cells are observed. CD44 is an activation marker and presumably cellular lesions result from more potent PEC activation as compared with the more sclerotic lesions (Kuppe et al. 2015). Not only CD44 but also markers constitutively expressed by PECs have been shown to be useful markers in the detection of early FSGS lesions in biopsies and can also be employed to differentiate between FSGS and minimal change disease (Smeets et al. 2014).

Taken together, the above findings strongly argue in favor of a single common cellular mechanism leading to extracapillary hypercellularity and ultimately to sclerosis in all glomerular disease, irrespective of the underlying cause. PEC activation and subsequent proliferation are the key elements. Presumably, the extent of PEC activation determines the cellularity of the extracapillary lesions and, thus, also the amount of fibrosis that will follow. This mechanism can be regarded as a glomerulus-specific “wound-healing” reaction and may even be adequate in certain circumstances. However, PEC proliferation often appears to be excessive and deleterious, especially in patients with diffuse CrGN and collapsing FSGS, hence the rationale for treating these conditions with aggressive immunosuppressive therapy. Pharmacological intervention in the process of PEC activation and proliferation is potentially of tremendous value for future patient care.

## Targeting PEC activation

**Fig. 2:**
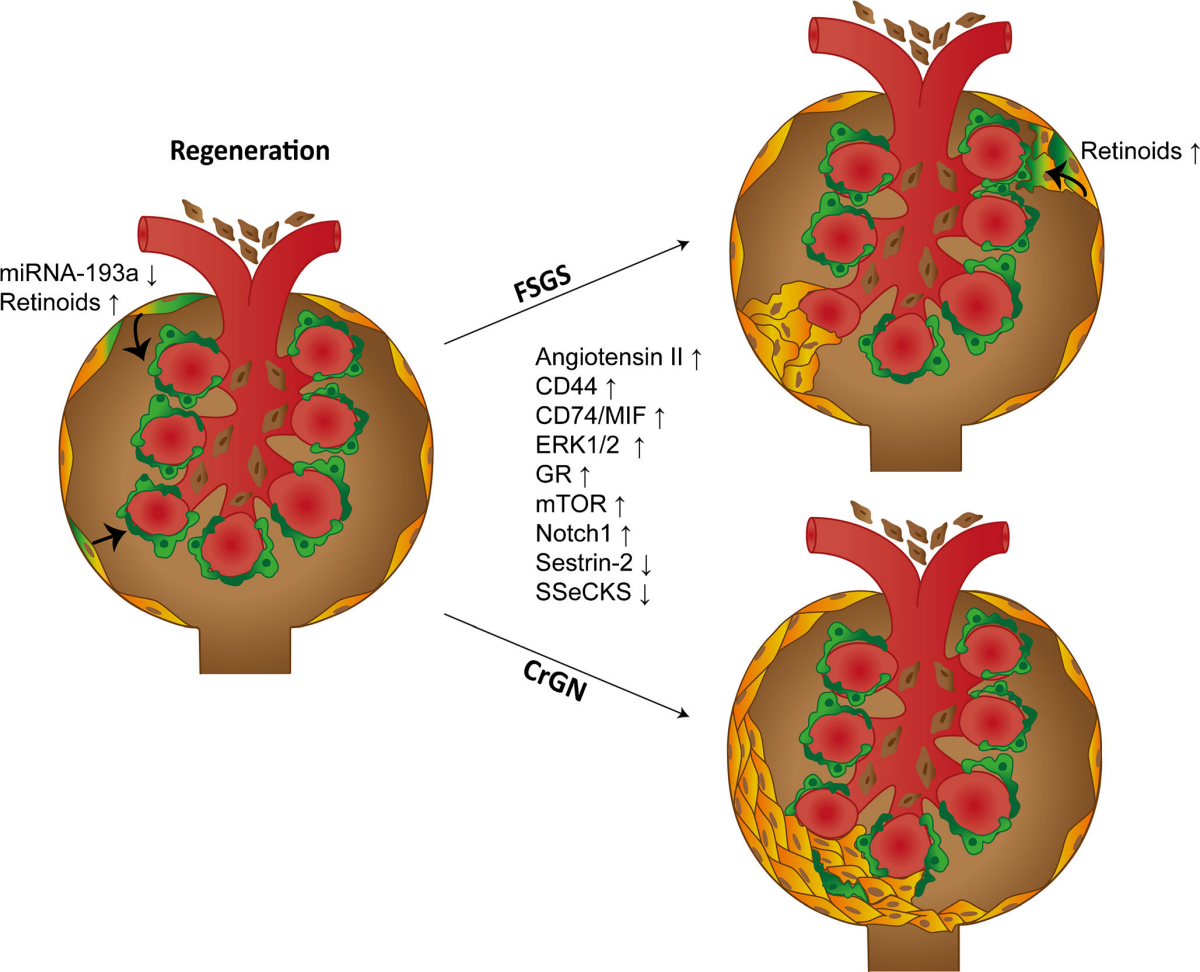
Representation of glomerular regeneration and glomerular disease (FSGS and CrGN) and factors that have been associated with these processes. *Vertical arrows* lying *behind* the factors indicate whether an up-regulation or down-regulation of the expression/activity was observed in the PECs. PECs are involved in FSGS and in the formation of crescents in CrGN. In FSGS, activated PECs (*orange* cells) migrate onto the glomerular capillaries and deposit matrix (sclerosis). In CrGN, the activated PECs form typical crescentic lesions. Recent studies have reported that, during disease regression, a subset of PECs at Bowman’s capsule or within a lesion on the capillary tuft differentiate towards a podocyte phenotype (*orange* to *green* cells, *arrows*)

The acknowledgement of the importance of PECs in the fate of the glomerulus has stimulated research investigating the molecular pathways associated with PEC proliferation, migration and extracellular matrix production (activation). Currently, several molecules have been identified as being differentially expressed in glomerular lesions and associated with PEC activation and/or transdifferentiation in human and experimental glomerular disease (identified molecules are listed in Table 1, Fig. 2).

**Table 1:** Summary of recent studies identifying molecules associated with activation of parietal epithelial cells (*PECs*)

Signaling factor and treatment	Effect on PECs	Model	Study reference
mTORC1 inhibition	Increased proliferation Increased PEC density	Mouse model of acute podocyte depletion Aging mice	McNicholas et al. 2016
mTORC1 inhibition or L-type neutral amino acid transporters inhibition	Cell necrosis Decreased crescent formation	Rats with crescentic glomerulonephritis (CrGN); WKY/NCrj rats injected with anti-glomerular basement membrane	Kurayama et al. 2011
Sestrin 2 reduction and increased mTOR activity (observation) Sestrin 2 inhibition in vitro	Associated with increased apoptosis or proliferation (dependent on the disease model) Increased apoptosis (in vitro)	Adriamycin and pruromycin nephropathy model in rat CrGN Conditionally immortalized mouse PEC cells	Hamatani et al. 2014
Reduced phosphorylated extracellular signal-regulated kinase 1 and 2 (p-ERK1/2) (observation)	PEC apoptosis	Mouse model of protein overload nephropathy Conditionally immortalized mouse PEC cells	Chang et al. 2012
Matrix metalloproteinase 9 increase and ERK activation (observation)	Possible increased migration and loss of adjacent podocytes	Zucker diabetic fatty rats Primary rat PECs	Zhang et al. 2015
Glucocorticoid receptor (GR) inhibition	Decreased PEC activation De novo expression of podocyte markers	Mouse model of acute podocyte depletion	Zhang et al. 2013
Cell specific GR inactivation and systemic GR activation and inhibition	Decreased proliferation and migration Decreased crescent formation	Pax8-Cre/GR^fl/fl^ mice with induced CrGN glomerulonephritis (NTN model)	Kuppe et al. 2016
Migration inhibitory factor (MIF) and CD47 deficiency MIF stimulation in vitro	Decreased PEC activation and proliferation Decreased crescent formation Induced PEC proliferation	NTN model in: MIF^-/-^ mice CD47^-/-^ mice Primary murine PECs	Djudjaj et al. 2016
Notch1 inhibition Notch1 inhibition in vitro	Reduced PEC proliferative lesions and reduced exspression of mesenchymal markers Decreased migration and mesenchymal transcript expression	NEP25 mice model for cFSGS Immortalized murine PEC cell line	Ueno et al. 2013
Interferon-α stimulation in vitro	Cell-cycle arrest and inhibition of migration of PECs	Immortalized murine PEC cell line	Migliorini et al. 2013
SSeCKS deficiency	Increased proliferation	NTN model in SSeCKS^-/-^ mice	Burnworth et al. 2012
Angiotensin II inhibition (via ACEi)	Decreased proliferation of PEC Less extracapillary lesions	MWF rats (spontaneous glomerulopathy)	Benigni et al. 2011

The findings of three different studies revealed that the mechanistic target of rapamycin (mTOR) signaling plays an important role in the physiology and phenotype of PECs and that changes in mTOR activity can trigger PEC activation or necrosis in experimental glomerular disease (Kurayama et al. 2011; Hamatani et al. 2014; McNicholas et al. 2016). mTOR is a serine-threonine kinase that constitutes a part of two distinct multiprotein complexes, namely TOR complex 1 (TORC1), which is sensitive to rapamycin and TORC2, which is not sensitive to rapamycin. In these studies in experimental models of CrGN and/or FSGS, mTOR signaling is increased in the glomerular epithelial cells involved within the lesions as shown by the increased phosphorylation of down-stream molecules p70 ribosomal protein S6 kinase (p70S6K) and 4E-binding protein 1 (4E-BP1), which stimulate ribosome biogenesis and translation to increase cell mass. In turn, phosphorylated (P-)p70S6K phosphorylates S6 ribosomal protein (S6RP), which also stimulates translation. S6RP has been shown to co-localize with podocyte markers and with PAX8 and CD44 expressed by PECs. The strong increase in mTOR activity in glomerular lesions and the general importance of mTOR signaling in cell survival growth and proliferation make it a possible therapeutic target to prevent or inhibit the development of glomerular lesions. However, McNicholas et al. (2016) demonstrated that the inhibition of mTORC1 with rapamycin does not benefit the glomerulus, as they observed an increase in the number of (severely) injured podocytes and activated PECs in a mouse model of acute podocyte injury. This results in an increase of extracapillary proliferative lesions and is also seen in kidney sections of aged mice treated with rapamycin. The unexpected worsening of the glomerular pathology through mTOR inhibition can possibly be explained by the onset, length, or strength of inhibition. mTOR is an essential regulator of cell metabolism, growth, proliferation and survival, indicating that an over-strong or -long inhibition leads to an aggravation of glomerular injury instead of healing as has been described in detail for podocytes (Godel et al. 2011). Moreover, the timing of inhibition of mTOR probably has an important role in PEC activation as demonstrated in the a study by Kurayma and coworkers (2011) showing that the early inhibition of mTOR in rats with CrGN leads to increased cellular necrosis of PECs, whereas later treatment (7 days after CrGN onset) reduces glomerular crescent formation. This finding illustrates that, within the same pathological process, mTORC1 inhibition can result in different outcomes. Despite the finding that the effects of mTOR inhibition diverges in the latter studies, they clearly link mTOR signaling to the activation of PECs and the development of glomerular lesions. Nevertheless, mTOR is essential in most, if not all, cells of the human body and rapamycin treatment will have effects far beyond those on the PECs and the glomerulus, complicating the interpretation of the above findings and also possibly complicating treatment.

A recent study by Hamatani et al. (2014) further connected mTOR signaling to PECs. Hamatani et al. (2011) showed that, in normal rat kidney sections, sestrin 2 is selectively expressed in PECs. Sestrin 2 is a member of a family of stress-inducible proteins that counteract oxidative stress. Specifically, sestrin 2 represses cell proliferation and growth by the inhibition of mTOR signaling. In contrast to other cell types in which sestrin 2 expression is related to stressors, normal adult PECs constitutively express sestrin 2. In other disease models, sestrin 2 expression decreases and mTOR signaling increases in PECs. In vitro studies with conditionally immortalized mouse PECs validated the converse correlation of sestrin 2 and mTOR activity, as the silencing of sestrin 2 leads to an increase in downstream targets of mTOR. However, the decrease of sestrin 2 and elevated mTOR activity result in different outcomes, i.e., PEC proliferation or PEC apoptosis, depending on the experimental disease. This study suggests the presence of a specific regulator of mTOR signaling within PECs.

Recently, Djudjaj et al. (2016) investigated the role of macrophage migration inhibitory factor (MIF) in the development of CrGN. MIF is a pleiotropic cytokine that mediates inflammation via its receptor CD74. CD74 signals within a complex involving the components CXCR2, CD44 and CXCR4, of which the two last-mentioned exhibit increased expression within activated PECs. Djudjaj et al. (2016) demonstrated that MIF, CD74 and CD44 are up-regulated in the glomeruli of patients and mice with proliferative glomerulonephritides. During disease, CD74 and CD44 are expressed de novo in PECs and are co-localized in PECs and mesangial cells. The secretion of MIF from glomerular cells and, in particular, from podocytes is increased. This induces the proliferation of PECs and mesangial cells in vitro. Both CD47- and MIF-deficient mice possess marked protection from glomerular injury, PEC activation and extracapillary proliferation. Hence, the study of Djudjaj et al. (2016) exhibited another new factor involved in PEC activation and pathological proliferation in glomerular disease.

Recently, Kuppe et al. (2016) studied the local actions of glucocorticoids in experimental glomerulonephritis. Glucocorticoids have become a standard therapeutic option for patients with nephrotic syndrome and are central in the treatment of most glomerulonephritides. The beneficial effects of glucocorticoids in these patients is commonly assumed to be attributable to a dampening of the immune response. However, in recent studies, local and non-immunological mechanisms of glucocorticoid action have been identified (Zhang et al. 2013; Kuppe et al. 2016). In the study by Kuppe et al. 2016), the glucocorticoid receptor (GR) was inactivated specifically in the kidney epithelial cells by using Pax8-Cre/GR^fl/fl^ mice. In Pax8-Cre/GR^fl/fl^ mice with induced CrGN, the genetic inactivation of GR results in reduced proteinuria, PEC activation and proliferation and, subsequently, in crescent formation. Interestingly, the beneficial effects of the genetic inactivation are similar to the effects observed in mice treated with high doses of prednisolone but without the immunosuppressive effects induced with prednisolone. This study indicates that both the stimulation of GR with prednisolone and its inactivation showed similar effects. This has further been demonstrated by using mifepristone, a partial GR antagonist. Pharmacologic treatment with mifepristone also attenuates disease as effectively as high-dose prednisolone but without the systemic immunosuppressive effects. By inhibiting the GR in kidney epithelial cells, Kuppe and colleagues (2016) demonstrated a direct effect of glucocorticoids on PECs. The use of glucocorticoid antagonists represents a novel therapeutic approach in CrGN with possibly less adverse effects compared with high-dose steroid treatment.

## Future perspectives

In the last decade, insights have been gained regarding the significant role of PECs in processes determining the fate of the glomerulus. Data are strong with regard to the involvement of PECs in the process of glomerulosclerosis but are however confounding regarding the involvement of PECs in podocyte regeneration. Therefore, future studies should provide more quantitative data on podocyte number in human kidneys to provide solid evidence for the existence of podocyte regeneration. PEC activation is a key process in the common pathways to glomerulosclerosis in glomerular disease. Although some pathways involved in PEC proliferation have been described, the processes mediating PEC activation remain unknown. Emphasis should be placed on the better characterization of the processes leading to PEC activation. For instance, this can be achieved by a comparison of the transcriptome and proteome of normal and activated PECs, possibly leading to the identification of initiating factors. The identification of such factors might then lead to the development of therapeutic interventions that prevent or attenuate PEC activation, both of which might be beneficial in many different progressive glomerular diseases.
